# High Structural Stability of Textile Implants Prevents Pore Collapse and Preserves Effective Porosity at Strain

**DOI:** 10.1155/2015/953209

**Published:** 2015-04-20

**Authors:** Uwe Klinge, Jens Otto, Thomas Mühl

**Affiliations:** ^1^Department for General, Visceral and Transplant Surgery, the University Hospital of the RWTH Aachen, Pauwelsstraße 30, 52074 Aachen, Germany; ^2^Department of Electrical Engineering and Information Technology, Aachen University of Applied Sciences, Eupener Straße 70, 52066 Aachen, Germany

## Abstract

Reinforcement of tissues by use of textiles is encouraged by the reduced rate of recurrent tissue dehiscence but for the price of an inflammatory and fibrotic tissue reaction to the implant. The latter mainly is affected by the size of the pores, whereas only sufficiently large pores are effective in preventing a complete scar entrapment. Comparing two different sling implants (TVT and SIS), which are used for the treatment of urinary incontinence, we can demonstrate that the measurement of the effective porosity reveals considerable differences in the textile construction. Furthermore the changes of porosity after application of a tensile load can indicate a structural instability, favouring pore collapse at stress and questioning the use for purposes that are not “tension-free.”

## 1. Introduction

Reinforcement of tissues by use of textile implants is increasingly used to improve the recurrence rates compared to unification of tissues just by sutures. However, at the occasion of revision operations it becomes apparent that the textile gets integrated into a tissue with more or less scar reaction. Whereas sometimes the implant is hardly palpable due to soft tissue reaction in other cases it was embedded in a thick and stiff scar plate. It is this excessive scar with consecutive contraction and thereby shrinkage of the mesh area that is related with most serious complications such as severe vaginal pain, dyspareunia, vaginal shortening, urethral obstruction, and SUI recurrence. Surgical intervention is often required to alleviate symptoms.

Scar formation can be stimulated by the trauma of surgery or by the presence of bacterial infection in the wound, but it can be stimulated by the implant as foreign body itself, even in the absence of excessive tissue damage or an infection. The local intensity of inflammatory and fibrotic tissue is significantly influenced by the configuration of the textile. In numerous studies it could be proven that the presence of large pores is mostly decisive for the quality of the tissue reaction [1].

The pore of a textile can be grasped as the area between filaments. Taking the area of the mesh fibre in comparison to the entire mesh area this results in a measurement for the “textile” porosity. However this measurement does not consider the geometry of the pores. High textile porosity can be achieved by many tiny pores (e.g., fleece) as well as few large pores. Whereas the textile porosity may be comparable the tissue reaction is not, as the latter is affected by the different contact surface and the different pore geometry. Only pores with a sufficient distance between the mesh fibres in all directions provide an area for recovery and local tissue regeneration in the centre. Correspondingly, small pore textiles even with less material but enhanced surface and lacking areas with sufficient distance to mesh fibres induce more inflammation than large pore materials made of more material of thicker monofilaments [2].

Small distances between fibres will result in linkage of foreign body reaction of opposite fibres and will promote filling the entire pore by scar, the so-called bridging. Experimental data showed that a critical minimum of a pore to diameter to prevent bridging is about 1 mm [3]. The area of a textile with pores with a diameter of less than 1 mm is supposed to show bridging, whereas the pores with a diameter of more than 1 mm, the so-called effective pores, are supposed to not show scar tissue within the pores but fat tissue. Thus the “effective porosity” of a mesh reflects its risk to get entrapped into scar and thus reflects biocompatibility in regard to tissue integration [4, 5]. In case of substantial attenuation of the inflammatory stimulus of the polymer surface it is conceivable that even smaller pores may be filled by local physiological tissues instead of getting filled up just by scar fibrosis.

In the 60s textiles have been introduced as being used “tension-free” compared to the tension that results from sutures, without considering any subsequent deformation by mechanical stress. The concept of “tension free” may still be reasonable for many procedures and in many parts of the abdominal wall or the groin. But meanwhile textiles are used to reinforce muscle plates of the diaphragm or the pelvic floor in areas that are suspected to put considerable mechanical stress on the textile implants [6, 7]. Correspondingly the tensile load leads to an elongation of the textile, which mainly results from deformation of the pore geometry. At mechanical stress the pores become elongated and narrowed, thereby reducing the distance between filaments and the effective porosity and increasing the risk for scar entrapment.

Since the remarkable studies by Petros et al. in the 90s pelvic floor prolapse as well as urinary incontinence is treated by stabilizing the pelvic floor with textile implants, often configured as slings to reinforce or replace defective ligaments [8, 9].

In the following study we analysed at two textile implants currently used as reinforcement of the pubourethral ligament for treatment of stress urinary incontinence in women whether mechanical strain changes the textile and effective porosity and thereby the predicted risk for scar entrapment after tissue integration.

## 2. Material/Methods

**Figure 1:**
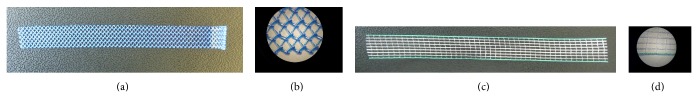
Sling device with the section being cut off for analysis. (a) TVT device textile part, (b) polypropylene filament and pore structure, (c) DynaMesh SIS soft textile part, and (d) PVDF filament and pore structure.

The mesh used was either a TVT from Ethicon (810041BL), which is cut off from large meshes (Prolene mesh in the version before 1998) that are made as textile hosiery of polypropylene monofilaments, or a DynaMesh SIS soft made of PVDF by the FEG Textiltechnik, Aachen (Figure 1).

**Figure 2:**
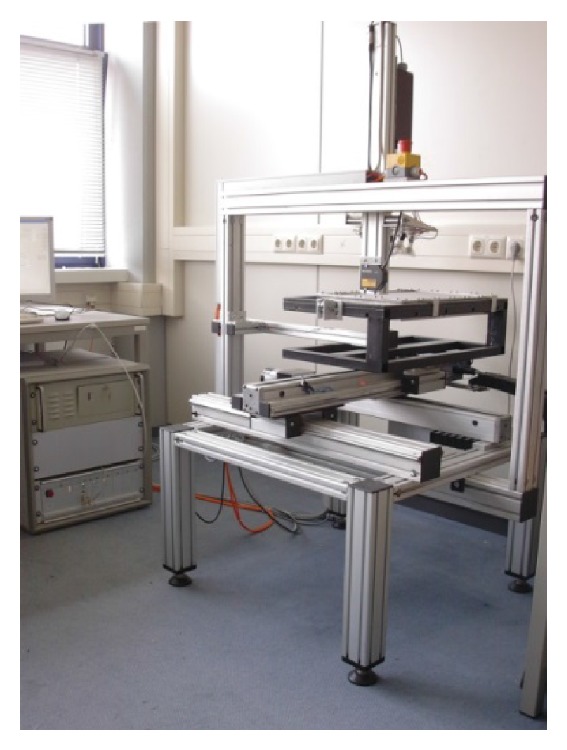
Experimental stand for the measurement of the effective porosity.

The porosity measurement system as described in [4] includes mesh fixing, position control, mesh illumination, camera system, control and evaluation unit, mechanical strain by weights, and evaluation software (Figure 2). For measurements without mechanical strain the mesh is fixed with magnets on an iron plate; for measurements with mechanical strain the mesh is fixed with clamps on both sides. The position control in two axes via step motors allows the selection of the measured part of the mesh. The mesh is illuminated from the back by a plane LED array with a diffusion glass. The camera system takes images from the top. Pixel resolution is 10 *μ*m × 10 *μ*m. During each measurement 6 images are taken. They are combined to one large image of the mesh. The evaluation is done by dedicated software named BKV-Standard, which is based on standard image processing tools. The image is converted into a black and white image via adaptive thresholds. Edge sharpening and noise reduction are done via digital filtering. Pores are detected and evaluated and finally the textile porosity and effective porosity of the mesh are determined. Due to the published experimental data a minimum distance between filaments of 1000 *μ*m for polypropylene and of 600 *µ*m for PVDF meshes is set for the calculation of the effective pore areas and the effective porosity [3].

The results of the porosity evaluation are as follows:

- *textile* porosity (percentage of area not covered by fibres in relation to the mesh area, irrespective of the geometric form),
- effective porosity (percentage of area that is filled only by sufficiently large (effective) pores in relation to the total mesh area),
- histogram of pore sizes (derived from square root of the pore area),
- histogram of* effective* pore sizes (derived from square root of the effective pore area, which is the area of the pore that is big enough to contain a sphere with a diameter larger than the critical limit).

Additional result is the measured total length of the mesh between the clamps for the determination of the elongation under force.

The measurement system is periodically calibrated with a perforated metal plate and the results are compared with mechanical measurements to ensure reproducible and reliable results.

## 3. Results

### 3.1. Polypropylene Sling (TVT)

**Figure 3:**
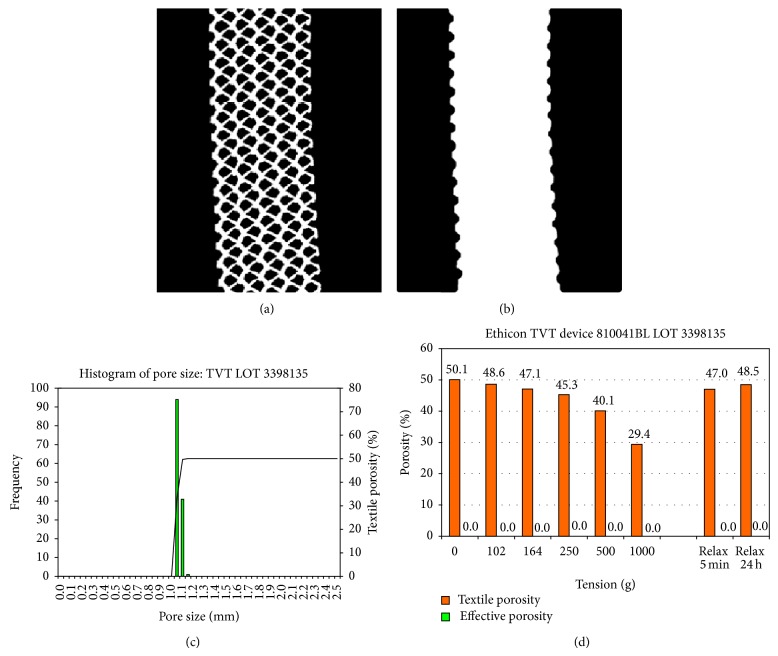
Image of TVT with (a) all pores, (b) effective pores, (c) pore frequency in dependency of pore size (estimated by simple square root of the pore area), and (d) textile and effective porosity at mechanical strain of up to 1000 g (8.9 N/cm).

The meshes are fixed with magnets on a metal plate without any forces applied. The textile porosity of the TVT is 50.1% whereas the effective porosity considering only large pores with a diameter of >1 mm to all sides is 0% (Figure 3). Due to the fact that several pores are just at the limit of 1000 *µ*m distance between filaments, the determined value for the effective porosity could be dependent on the area, where the sample is measured, and is sensible to smaller changes in mesh production and sample handling.

Repetition of the measurements 5 times resulted in a mean value of 50.2% ± 0.24% for the textile porosity and 0.0% ± 0.0% for effective porosity and confirmed the reproducibility of the procedure. 24 h after release of the strain textile porosity recovers to 48.5%, but a slight elongation of 6.8% still persists.

**Figure 4:**
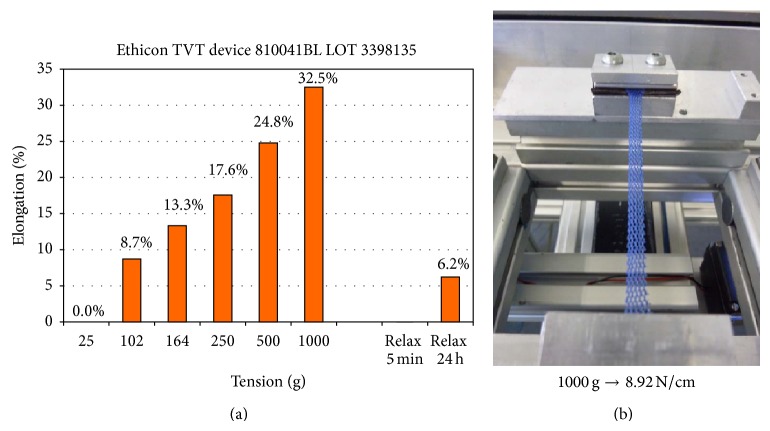
Elongation of the TVT at mechanical load as percent of original length (a) with macroscopic image (b).

Measurements with application of tensile forces of 0.9 to 8.9 N/cm at the TVT sling with the width of 11 mm led to a reduction of the textile porosity to 29.4% at a load of 8.9 N/cm, whereas the effective porosity always was 0%. This corresponds to an elongation of up to 32.5% due to deformation of pore geometry (Figure 4).

### 3.2. PVDF Sling (SIS)

**Figure 5:**
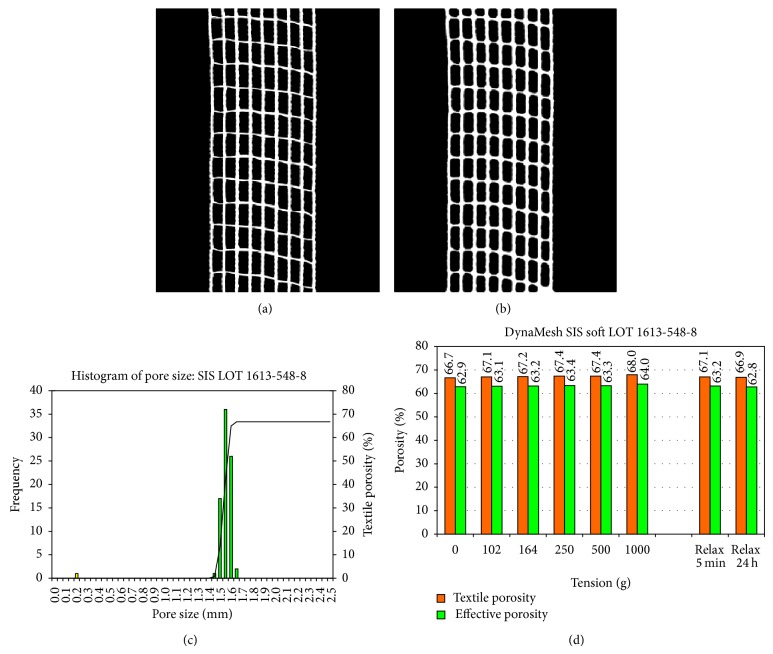
Image of SIS with (a) all pores, (b) effective pores, (c) pore frequency in dependency of pore size (estimated by simple square root of the pore area), and (d) textile and effective porosity at mechanical strain of up to 1000 g (8.9 N/cm).

The textile porosity of the TVT is 66.7% whereas the effective porosity considering only large pores with a diameter of >0.6 mm to all sides is 62.9% (Figure 5). Repetition of the measurements 5 times resulted in textile porosity in a mean value of 66.4% ± 0.22% and confirmed the reproducibility of the procedure. 24 h after release of the strain textile porosity was still constant with 66.9% and only a very little elongation of 0.4%.

**Figure 6:**
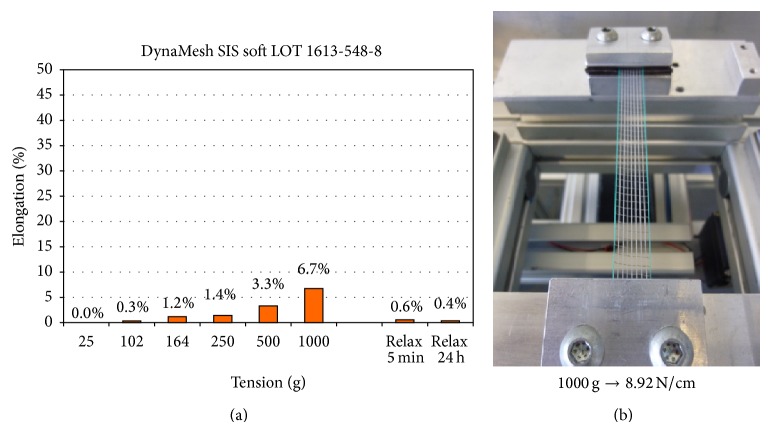
Elongation of the SIS at mechanical load as percent of original length (a) with macroscopic image (b).

Measurements with application of tensile forces of 0.9 to 8.9 N/cm at the SIS sling with the width of 11 mm led to a slight increase of the textile porosity to 68.0% at a load of 8.9 N/cm and of the effective porosity to 64.0%. This corresponds to an elongation of 6.7% due to deformation of pore geometry (Figure 6).

## 4. Discussion

Measurement of the effective porosity at mechanical strain reveals differences of the textile construction with important consequences for the risk of scar entrapment after tissue integration. Measurement of the textile porosity obviously at rest is not sufficient to predict the changes of pore geometry at tensile load.

The TVT sling implant shows acceptable textile porosity but an absence of large or effective pores, indicating for most of the mesh area a high risk for getting completely surrounded by fibrotic scar tissue. Although already without application of any forces the mesh showed an insufficient pore size, the pore sizes are dramatically reduced further when mechanically stressed. Due to the collapse of the pores the mesh showed a considerable elongation with a narrowing in width leading to roping and curling of the textile. Similar stress may occur during implantation or as a consequence of the mobility of the pelvic floor.

In contrast the alternative design of the SIS sling showed a higher textile porosity, which is not compromised at tensile load. Almost all pores fulfil the criteria of effective pores resulting in a high effective porosity of more than 60% even at a strain of 1000 g! Correspondingly the device shows a restricted elongation reflecting the enormous structural stability.

As a result of the missing effective porosity the TVT has a higher risk for scar formation at the entire area of the mesh. Furthermore scar usually showed a contraction of at least 20%, and thus an implant being incorporated mainly in scar tissue will show an increased shrinkage of the mesh area with an implant getting folded and wrinkled. Indeed at numerous explanted slings from humans we could confirm the predominance of scar tissue around the TVT with shrinkage and folding in almost all specimens (unpublished data).

As demonstrated with the SIS, structural stability with high resistance to mechanical loads can be realised by choosing an adequate textile construction with tight binding and fibres running in line with the mechanical load. The present study with measurements of effective porosity under strain confirms the conclusion of Petros and Papadimitriou of a nonstretch tape to minimize obstruction and urethral damage [8]. And it provides a reasonable explanation that inelastic slings can be used with a lowered risk for mesh exposure [10]. Considering the considerable similarity of the textile structure of the slings from BARD, Gynecare, Caldera, AMS, and Boston Scientific it is not surprising that all these devices are subject to litigations for comparable problems [11]. Only the sling from Mentor shows a construction like the SIS sling, though with smaller pores.

In this study the maximum physiological strain for the abdominal wall was estimated to be less than 16 N/cm [12]. Tensile measurements of tissues revealed that the layers will hardly keep more than this before disintegration [13–15]. Thus for the area of the pelvic floor both theoretical calculations of the strain to be assumed as well as the maximum holding capacity of the tissues will put a limit of <10 N/cm to any mechanical strain. Janda even found that a membrane tension of 2 to 5 N/cm as strain should be expected in the pelvic floor, even only 1 N/cm in nonprolapsed tissues [16]. The tensile strain in the pelvic floor is expected to lead to an elongation of the textile. An elongation of up to 20% is considered to form the comfort zone, and elongation of 40% defines the safety zone [17]. Thus in regard to both force and elongation, our setting should widely reflect the physiological range. However for other indications for other devices in other areas of the body the mechanical strain should be adapted correspondingly.

In the current study the mechanical strain was applied as uniaxial testing. In this specific case it reflects an application of the textile intended to support or replace a ligament, for example, the pubourethral ligaments in case of slings for treatment of stress urinary incontinence. In this setting uniaxial testing should be regarded as acceptable. However looking at the properties of bigger mesh areas in a two-dimensional setting the compliance of the textile differs considerably in dependency of the mesh orientation [18–20]. In line with the fibres (machine direction) the stretchability is more restricted than perpendicular to this. Overall, uniaxial testing thus cannot be compared to the results of a multiaxial testing or a test pressing through the stamp.

Unfortunately multiaxial testing as with the ball burst test or the test pressing through a stamp is strongly influenced by the size of the sample. But even more for this testing the mesh is fixed at its borders so that any elasticity is restricted to the little elongation that is permitted by stretching of the fibres in the binding. Any huge deformation of the pore is inhibited by this fixation, and the resulting tension forces are significantly higher compared to the uniaxial testing and thus not comparable. Maybe in the future computer simulation by use of finite elements may help to grasp the anisotropic characteristics of textile implants. The current experimental measurements do not. Therefore testing conditions have to be clearly outlined if textiles are compared.

The in vitro investigation of the pore sizes and its changes at mechanical load helps to predict tissue response after implantation, in particular the extent of scar formation and whether this scar entraps the entire implant [5, 18]. The increased risk for fibrotic bridging has been confirmed for small pore devices used in the abdominal wall as well as in the pelvic floor. However, though superior tissue integration of textiles with large pores has been proven for the abdominal wall in many clinical studies, due to the lack of explanted slings from humans the specific tissue reaction to SIS still has to be shown.
